# Editorial: Advanced Bioremediation Technologies and Processes for the Treatment of Synthetic Organic Compounds

**DOI:** 10.3389/fbioe.2021.721319

**Published:** 2021-09-29

**Authors:** Kunal R. Jain, Chirayu Desai, Eric D. van Hullebusch, Datta Madamwar

**Affiliations:** ^1^ Post Graduate Department of Biosciences, Sardar Patel University, Anand, India; ^2^ P. D. Patel Institute of Applied Sciences, Charotar University of Science and Technology, Changa, India; ^3^ Institut de Physique du Globe de Paris, Université de Paris, Paris, France

**Keywords:** xenobiotics, bioreactors, bioremediation, biotoxicity, biodegradation

Synthetic organic compounds (SOCs) are the primary pollutants of aquatic and terrestrial ecosystems. This group of chemicals includes pesticides, herbicides, and pharmaceutical products and their transformed products; industrial chemicals (such as plasticizers, dyes, and dye intermediates), organophosphate and brominated flame retardants, volatile organic compounds (VOCs) (i.e., organic solvents), polycyclic aromatic hydrocarbons, per- and polyfluoroalkyl substances (PFAS), plastic polymers, and many more (Tijani et al., 2016; Ahmed et al., 2018; Ilyas and van Hullebusch, 2020). The SOCs are commonly found in urban wastewater, agricultural runoffs, and industrial waste streams (Postigo and Barceló, 2015). SOCs pollute various habitats through diffusion or as point source pollutants. SOCs exert multi-dimensional effects in the polluted environment by altering the physicochemical properties of the habitats where they are released, representing a threat to humanity (Naidu et al., 2021). The environmental distribution and persistence of many SOCs were previously unknown, but with the development of newer detection methods, SOCs have been found in the environment at much higher concentrations. Due to their bio-toxic properties, many SOCs are now recognized as priority pollutants, requiring immediate solutions for their removal from polluted ecosystems (Lohmann et al., 2007; Walker, 2019).

Soon after realizing the adverse effects of SOCs on the biosphere, different treatment technologies and processes, including hybrid integrated systems, have been developed. Due to the exponential rise in the generation of SOCs, and concomitant heterogeneous compositions of these xenobiotics in the polluted environment, traditional treatment technologies were found to have varying degrees of efficacy. The accumulating volume and heterogeneity of the xenobiotic wastes that are generated are the driving force for the emergence of next-generation technologies in waste treatment. In recent years, promising multi-dimensional treatment technologies have been developed such as hybrid integrated treatment systems coupling biological and physicochemical processes, membrane bioreactors, microbial electrochemical technologies, phyto-reactors, *in-situ* treatment methods (e.g., bioventing, biostimulation, and bioaugmentation), novel pre-treatment approaches, and innovative bioreactor/bioprocess designs for treatment of SOCs (Jain et al., 2020; Trellu et al., 2020).

Therefore the aim of this Research Topic is to collate the recent advances in the development of bioremediation processes and technologies for the treatment of synthetic organic compounds (SOCs). This research topic has published thirteen articles including six original studies, one brief research report, and six review articles (including one systematic review paper).

In their review, Mishra et al. explain the remarkable role of microorganisms and their catabolic potential, with genes, enzymes, and degradation pathways, in the biodegradation process of xenobiotic compounds. The successful execution of omics technologies with bioinformatics tools shows the way to next-level research in the bioremediation potential of microorganisms and exploits their capability to remove xenobiotic contamination.

Another review article (by Arora) described the competence of the genus *Bacillus* for the degradation of xenobiotic compounds along with the detoxification of potentially toxic metals. The author has listed 154 species of *Bacillus* and reported their use in the biodegradation and biotransformation of various pesticides, herbicides, and insecticides along with chlorophenol, nitrophenol, and chloronitrophenol, the decolorization and biotransformation of dye compounds, the biodegradation of different polycyclic and heterocyclic aromatic hydrocarbons, explosives, drugs, natural aromatic compounds, and other xenobiotic compounds like crude oils, plastic bags, etc., and the detoxification of potentially toxic metals. Several advanced technologies, like genome-editing tools used in bioremediation, have been also discussed. The author concludes that the construction of a genetically engineered Bacilli strain or genomic editing tool will help in developing efficient *Bacillus* strains for bioremediation.

Surkatti et al. studied the organic degrading bacteria from gas-to-liquid process water (GTL). They have isolated and identified the native bacteria from GTL process water, which were further used for biodegradation of organic contaminants from local GTL process water. The authors have used techniques from three areas of environmental microbiology and biochemistry along with bioremediation. Three distinct bacterial species namely, *Alcaligenes faecalis*, *Stenotrophomonas* sp. and *Ochrobactrum* sp. were identified. They observed that the bioremediation potential of mixed bacterial strains was much better than the three individual strains, though individually the treatment efficiency reached up to 60% reduction in chemical oxygen demand (COD).

Mahapatra and Phale provide insights into the ecological aspects of field application and microbial strain optimization for efficient bioremediation of polycyclic aromatic hydrocarbons (PAHs) following a system biology approach. In another review by Shahsavari et al. the chemical properties, sources, and fate of per- and polyfluoroalkyl substances (PFAS) contamination in the environment are discussed. This review systematically addresses the current status of PFAS biodegradation as well as challenges faced in their remediation.

Plastics or Low-Density Polyethylene (LDPE) have been one of the major global pollutants for last the several years. Due to its highly recalcitrant properties, its natural turnover rate in the environment is negligible. In an experimental study, Dey et al. demonstrated the degradation of LDPE using an enriched microbial community. The culturable portion of the community consists of *Stenotrophomonas* sp. and *Achromobacter* sp. The microbial growth in presence of LDPEs, high cell surface hydrophobicity of the microbial community, and substantial weight reduction of the treated LDPEs were the primary indicators that signified plastic degradation.

Maurya et al. reviewed the current research on enzymatic remediation of Polyethylene Terephthalate (PET) based polymers. The review provides a brief overview of the various approaches of biocatalysis used for effective recycling of PET, along with the factors affecting the hydrolysis rate and the challenges faced during this step. The authors suggest that further research should be focused on identifying thermostable PET-hydrolyzable enzymes capable of hydrolyzing hcPET with broad substrate specificity.

Dhakar et al. have demonstrated the construction of a genome-scale metabolic model following automatic and manual protocols and its application for improving metabolic potential through interactive simulations. In an experimental study, Ferreira et al. studied the phytoremediation efficiency of four plant species for soil samples contaminated with Tebuthiuron (herbicides) and the effect of vinasse during the treatment.

In a review by Jaiswal et al. SOC management from paper mill/pulp industrial effluents is discussed in detail. Authors have described various SOCs present in pulping and paper processing effluents. The review describes various methods to reduce SOC production from pulp and paper industries, and their treatment strategies were also discussed in brief along with their challenges and limitations. It further describes the biotechnological applications such as genetically modified biological agents for sustainable remediation of SOCs from paper industries.

In a brief research report by Mazioti et al. treatment of real bilge wastewater with zero valent iron (ZVI) and activated charcoal using three different biological methods: (i) anaerobic digestion with granular sludge and ZVI addition for enhancement of methane production, (ii) activated charcoal addition to biological treatment (aerobic and anaerobic) for significant reduction of COD, and (iii) combination of ZVI and anaerobic charcoal addition for high-performance treatment. The study found that a combination of ZVI and activated charcoal could have greater bioremediation performance amongst the three approaches used for the treatment of real bilge wastewater.

In a research article on total petroleum hydrocarbon (TPH) degradation, Garousin et al. have accessed various methods of bioremediation (i.e., bio-stimulated microcosm, bacterialized microcosm, combinatorial approach of bio-stimulated microcosm and bacterialized microcosm, and natural attenuation) using *Bacillus altitudinis* HRG-1 in age-old petroleum contaminated soils. The study reports that a combination of bio-stimulated microcosm and bacterialized microcosm was the most effective method, where 38.2% TPH was reduced under experimental conditions over a 60-day period. The author concludes that biostimulation alone was insufficient, and that proper application of suitable microbes is essential to reduce the contaminant load from polluted soils.

Askri et al. through their experimental study, demonstrated that bioelectricity can be generated during the treatment of real textile effluents using microbial halothermotolerant bioanodes from hypersaline sediment and textile dyeing wastewater. The authors claimed to produce a reproducible bioelectric current of about 12.5 ± 0.2 A/m^2^ with a simultaneous reduction in COD with 91 ± 3% efficiency.

This research topic highlights the microbial degradation of various SOCs by different approaches, including integrated methods. The technologies reported include traditional bioremediation approaches using pure microbial cultures as well as mixed cultures, integrated biological treatment, and advanced phytoremediation approaches, as well as genomics and metabolic studies to understand the biodegradation of SOCs. This research topic also discusses the current status of the research advances in the treatment of SOCs, the limitations and challenges for effective removal of SOCs, and offers a direction for future research in achieving optimal biotechnological solutions in the treatment of SOCs.
